# The effect of selenium supplementation on oxidative stress, clinical and physiological symptoms in patients with migraine: a double-blinded randomized clinical trial

**DOI:** 10.3389/fnut.2024.1369373

**Published:** 2024-05-02

**Authors:** Arghavan Balali, Omid Sadeghi, Fariborz Khorvash, Mohammad Hossein Rouhani, Gholamreza Askari

**Affiliations:** ^1^Student Research Committee, Isfahan University of Medical Sciences, Isfahan, Iran; ^2^Department of Community Nutrition, School of Nutrition and Food Science, Nutrition and Food Security Research Center, Isfahan University of Medical Sciences, Isfahan, Iran; ^3^Neurology Research Center, School of Medicine, Isfahan University of Medical Sciences, Isfahan, Iran

**Keywords:** selenium, antioxidant, oxidative stress, migraine, mood, clinical trial

## Abstract

**Background and Aim:**

The present double-blinded randomized clinical trial aimed to investigate the effect of selenium supplementation on oxidative stress, clinical, and physiological symptoms in patients with migraine.

**Methods:**

In total, 72 patients with migraine were randomly assigned to receive either 200 μg/day selenium (*n* = 36) or placebo (*n* = 36) for 12 weeks. Clinical traits of migraine (e.g., severity, frequency, and duration of headaches), mental health indices (e.g., depression, anxiety, and distress), quality of life, biomarkers of oxidative stress (e.g., nitric oxide [NO], malondialdehyde [MDA], total antioxidant capacity [TAC], total oxidant status [TOS]), and anthropometric indices were assessed at baseline and at the end of the study.

**Results:**

Selenium supplementation resulted in a significant reduction in NO (−1.24 ± 0.43 vs. 0.16 ± 0.43; *p* = 0.03) levels and a significant increase in TAC (9.89 ± 2.50 vs. −0.18 ± 2.50; *p* = 0.01) compared to the placebo group. Moreover, selenium supplementation had a significant protective effect against MDA levels compared to placebo (0.33 ± 0.57 vs. 1.83 ± 0.57; *p* = 0.03). In addition, selenium intake was associated with a lower headache frequency (−8.15 ± 0.77 vs. −4.12 ± 0.77; *p* < 0.001) and severity (−2.89 ± 0.42 vs. −1.16 ± 0.42; *p* = 0.01) as well as a lower Headache Impact Test-6 (HIT-6) score (−9.22 ± 2.00 vs. −2.08 ± 2.00; *p* = 0.02) compared to the controls. For other outcome variables, we found no significant effect.

**Conclusion:**

Selenium supplement may be considered a complementary therapy in patients with migraine due to its beneficial effects on oxidative stress and migraine symptoms. Further studies are needed to affirm our findings.

**Clinical Trial Registration**: This study was registered in the Iranian Registry of Clinical Trials (https://www.irct.ir) on 27 May 2023 with code number of IRCT20121216011763N60.

## Introduction

Selenium is an essential component of selenoproteins and antioxidant enzymes, which have drawn particular interest for their key role in neurological wellbeing (1, 2). A growing body of experimental research suggests that selenium is important for the brain, as the turnover rate of neurotransmitters is altered in selenium deficiency (3). According to this, the beneficial effect of selenium supplementation on some neurodegenerative diseases such as epileptic seizure (4) and cognitive disorder (5) has been established. However, the effect of selenium on migraine is unclear. A recent case–control study in Iran correlates migraine headaches with reduced serum levels of selenium (6). Moreover, a cross-sectional study revealed that low dietary intake of selenium (less than 93.1 μg/day) is associated with lower odds of migraine headaches (7).

Migraine is a prevalent, disabling neurological disorder with a prevalence of 14–15% worldwide (8) and 15.1% in Iran (9). Migraine is characterized by moderate-to-severe, unilateral, and throbbing headaches, which often last between 4 and 72 h, along with photophobia, phonophobia, vomiting, and nausea (10). The Third Edition of the International Classification for Headache Disorders (ICHD-3) categorized migraine into two major subtypes: migraine with aura and migraine without aura (11). Aura is a transient sensory disturbance that occurs before or at the beginning of migraine attacks (11). Aura symptoms may contain temporary disturbances in the visual such as blind spots, flashes of light, other vision changes, or tingling in the hand/face.

Despite the complex nature of migraine, some pieces of evidence suggest that oxidative stress is involved. Therefore, several antioxidant supplements have been proposed for the management of migraine symptoms (12, 13). Prior studies have shown that some antioxidants including magnesium (14), zinc (15), coenzyme Q10 (16), and vitamin C (17) have beneficial effects on oxidative stress and migraine symptoms. However, to the best of our knowledge, no clinical trial has investigated the effect of selenium supplementation on migraine outcomes. Considering the antioxidant and anti-inflammatory effects of selenium, we hypothesized that selenium might be beneficial for the management of oxidative stress and clinical symptoms in migraineurs. Thus, the present double-blinded randomized clinical trial aimed to examine the effects of selenium supplementation on oxidative stress, clinical and physiological symptoms, and quality of life in a sample of patients with migraine.

## Materials and methods

### Participants and study design

The current investigation was a parallel, double-blinded, randomized placebo-controlled clinical trial. Participants were recruited from the neurology clinic of the Al-Zahra Hospital affiliated with the Isfahan University of Medical Sciences, Isfahan, Iran, between May and August 2023. More details regarding study design and participants have been published previously (18). Migraine headache was confirmed by a neurologist according to the ICHD-3 criteria (11) at least in the last 6 months. Individuals were included in this study if they: (1) had a confirmed diagnosis of migraine according to the ICHD-3 criteria, (2) were between 18 and 65 years, and (3) had at least two attacks per month. Individuals who: (1) were pregnant or lactating; (2) consumed antioxidant supplements (e.g., multivitamins, vitamin C, and omega 3) in the last 3 months; (3) had a medical history of infectious disorders (e.g., coronavirus and hepatitis), autoimmune disorders, diabetes mellitus, cardiovascular diseases, and other neurological disorders such as other subtypes of headaches (i.e., tension-type headache), epilepsy, Parkinson’s disease, and multiple sclerosis; and (4) were current smokers were not included in the study. Patients were excluded if they: (1) suffered from complications related to selenium supplementation; (2) had changed the type or dosage of their medications during the intervention; and (3) were not willing to continue the intervention.

### Ethical consideration

Each participant provided written informed consent before the trial. The ethics committee of Isfahan University of Medical Sciences has approved the study protocol (IR.MUI.RESEARCH.REC.1401.404). Moreover, this clinical trial was registered in the Iranian Registry of Clinical Trials^1^ with the code number IRCT20121216011763N60. The study protocol was in accordance with the Declaration of Helsinki, and all findings were reported according to the Consolidated Standards of Reporting Trials (CONSORT) guidelines (19).

### Sample size

Given the type I error of 5% (α = 0.05) and the type II error of 20% (*β* = 0.20, power = 80%), the required sample size was calculated using the following formula (20). Based on a prior study (21), mean differences (±SD) of malondialdehyde in the intervention and control groups were − 0.13 ± 1.03 and 1.4 ± 3.11, respectively. By considering the abovementioned information, a minimum sample size of 33 was needed in each intervention group. However, by considering a 10% drop-out, 36 patients with migraine were included in each arm.


$$n=\frac{2{\left(a+b\right)}^{2}\;\sigma^{2}}{{\left(\mu 1-\mu 2\right)}^{2}\;}$$

### Randomization

After the recruitment, stratified block randomization was performed based on participants’ age (18 to 39 and 40 to 65 years) and sex (male or female) across the two groups using a four-block size. Then, within each of the aforementioned strata, eligible participants were randomly assigned in a 1:1 ratio to receive either selenium or placebo. Balanced randomization was generated using an online tool^2^ by a person who was not involved in the study. Investigators and participants were blinded to the treatment allocation.

### Intervention

Patients in the selenium group (*n* = 36) received a tablet containing 200 μg selenomethionine per day prepared by Pourateb Company under the license of Nature Made Company, United States. Previous studies have indicated that selenium supplementation of 200 μg per day was safe and helpful for the management of oxidative stress among other populations (21, 22). Patients in the placebo group (*n* = 36) received a tablet containing 200 μg of starch daily prepared by the Laboratory of Pharmaceutical Technology of the Faculty of Pharmaceutical Sciences of the Isfahan University of Medical Sciences (Isfahan, Iran). The selenium and placebo tablets looked identical, having the same shape, color, and size. Furthermore, routine care and treatment were provided by the neurologist. The trial lasted for 12 weeks. Consumption of supplements was monitored by phone call every 2 weeks. Participants were asked not to change their lifestyle, dietary habits, and medicines during the study. Compliance was calculated using the following formula:

Compliance rate = (tablets taken / tablets prescribed) × 100

### Assessment of dietary intake and physical activity

In total, six one-day dietary recalls (4 weekdays and 2 weekend days) were gathered from all participants during the study. Subjects were asked to report their dietary intakes based on household measures. Then, these reports were converted to grams using standard protocols (23). To calculate dietary intakes throughout the trial, we used the United States Department of Agriculture (USDA) nutrient databank adapted for Iranian foods. In addition, two one-day physical activity (PA) records (including a working day and a non-working day) were collected from all participants during the investigation. Levels of PA were reported as metabolic equivalent hours per day (METs/h/day).

### Anthropometric measures and blood pressure

Body weight was measured to the nearest 100 g using a digital scale (Omron BF511 (Omron Corp, Kyoto, Japan)) with participants wearing minimum clothing and no shoes. Using a non-stretched tape with an accuracy of 1 mm, height was assessed in a standing position with no shoes. Waist circumference (WC) was measured to the nearest 1 mm after a normal exhalation, at the midway between the lowest rib and iliac crest, with the participant in a standing position. To assess body mass index (BMI), weight in kilograms was divided by height squared in meters. Blood pressure (BP), including systolic and diastolic BP, was assessed using a digital sphygmomanometer (OMRON, M3, HEM-7154-E, Japan) after 10 min of seated rest. The measurements were taken twice, and the average value was reported as the final BP. All measurements were performed by the principal investigator (AB).

### Biochemical assessment

At the baseline and at the end of the study, a 5-ml blood sample was obtained from all participants. These samples were immediately centrifuged to separate the serums and then maintained at −80°C for future tests. In this study, the levels of nitric oxide (NO; Kiazist Life Sciences, Iran, catalog no: KNO96), total antioxidant capacity (TAC; Kiazist Life Sciences, Iran, catalog no: KTAC96), total oxidant status (TOS; Kiazist Life Sciences, Iran, catalog no: KTOS96), and malondialdehyde (MDA; Kiazist Life Sciences, Iran, catalog no: KMDA96) were measured as indicators of oxidative stress. The level of these indices was evaluated via the ELISA reader BioTek ELx800 (BioTek Instruments Inc., Winooski, United States) according to the protocol of the ELISA kits (Kiazist Life Sciences, Iran).

### Migraine assessment

Migraine characteristics including severity, frequency, and duration of migraine attacks were assessed by an experienced neurologist using a validated questionnaire. Headache severity was assessed using a visual analog scale (VAS) questionnaire on a 0–10 scale in which 0 represents “no pain” and 10 represents “the worst imaginable pain” (24). Patients noted the feeling that represents their perception of pain. The frequency and duration of migraine headaches were evaluated as the number of attacks per month and the mean of attack duration in a day, respectively.

### Assessment of quality of life

A growing body of evidence suggests that the quality of life of migraineurs is impaired (25). Therefore, we used two valid and reliable questionnaires including the Headache Impact Test-6 (HIT-6) and the Migraine-specific Quality of Life (MSQ) to investigate the effect of selenium supplementation on the quality of life of migraineurs.

The HIT-6 questionnaire focuses on the effect of headaches on the wellbeing and daily performance of patients with migraine (26). This questionnaire includes six items. Each item has five response options, scored between 6 and 13, including never (six points), rarely (eight points), sometimes (10 points), very often (11 points), and always (13 points) options. Therefore, the total score of HIT-6 ranges from 36 to 78 and is classified into four categories: 36–49, 50–55, 56–59, and ≥ 60 as no, moderate, substantial, and severe impact of headaches on patients’ quality of life, respectively. The validity and reliability of this questionnaire had been approved previously (27).

The MSQ (version 2.1) investigates the effect of migraine headaches on patients’ daily functioning by considering the past 4 weeks (28). This questionnaire has three domains: (1) the Role Restrictive (RR) domain describing the reduction of daily activities (7 items), (2) the Role Preventive (RP) domain that explains the influence of migraine headaches on normal work and social activities (4 items), and (3) the Emotional Functioning (EF) domain that evaluates the emotions associated with headache (3 items). Each item includes six response options, scored between 1 and 6: none of the time (score 1), a little bit of the time (score 2), some of the time (score 3), a good bit of the time (score 4), most of the time (score 5), and all of the time (score 6). The total score of each domain is computed as a sum of item responses and rescaled from 0 to 100, with higher scores indicating a better quality of life.

### Mental health assessment

For the assessment of psychological disorders, we used a validated 21-item Depression, Anxiety, and Stress Scale (DASS-21) (29). This questionnaire consists of seven questions for each subscale of depression, anxiety, and distress with a total of 21 questions. Each question can be responded to with four options, ranging from 0 (does not apply to me at all) to 3 (applies to me most of the time). The total score for each subscale of DASS-21 ranges from 0 to 21, with higher scores representing higher levels of psychological distress. To interpret scores on the same scale as DASS-42, the total score for each subscale was multiplied by two (30).

### Assessment of other variables

All participants completed a general questionnaire including age, sex, marital status (single/married), education (lower than diploma/diploma and higher), medical history (anemia, hypo/hyperthyroidism, respiratory disorders, kidney, and liver diseases), medicine history (propranolol, alventa, topiramate, gabapentin, chlordiazepoxide, pantoprazole, and amitriptyline), migraine in first-degree relative (yes/no), and supplementation (vitamin D, magnesium, and riboflavin) at the beginning of the study. Moreover, the socioeconomic status (low/intermediate/high) of subjects was evaluated through specifically designed questions such as the number of family members, home ownership, car ownership, and the participant’s job, using a face-to-face interview.

### Statistical analysis

The analyses were performed on the basis of the intention-to-treat (ITT) approach. The Kolmogorov–Smirnov test was applied to examine the normal distribution of outcome variables. To normalize the non-normally distributed variables, a log transformation was conducted. Quantitative variables were expressed as mean ± SD, and qualitative variables were represented as number (percent). An independent sample *t*-test was performed to detect differences in quantitative variables between the selenium and placebo groups. For comparing the qualitative variables between the two groups, the chi-square test was performed. Furthermore, a one-way analysis of covariance (ANCOVA) was applied to detect the effect of selenium supplementation on outcome variables by controlling for potential confounders. In this analysis, baseline values of outcome variables, energy intake, vitamin C, and medication with serotonin and norepinephrine reuptake inhibitors (SNRIs) were adjusted. Moreover, mean differences in the changes in outcome variables during the intervention period between the two groups were compared by ANCOVA. *p*-values of all statistical analyses were conducted using the SPSS software version 21 (SPSS, Inc. Chicago, IL, United States). *p*-values less than 0.05 were considered statistically significant.

## Results

### Participants

**Figure 1:**
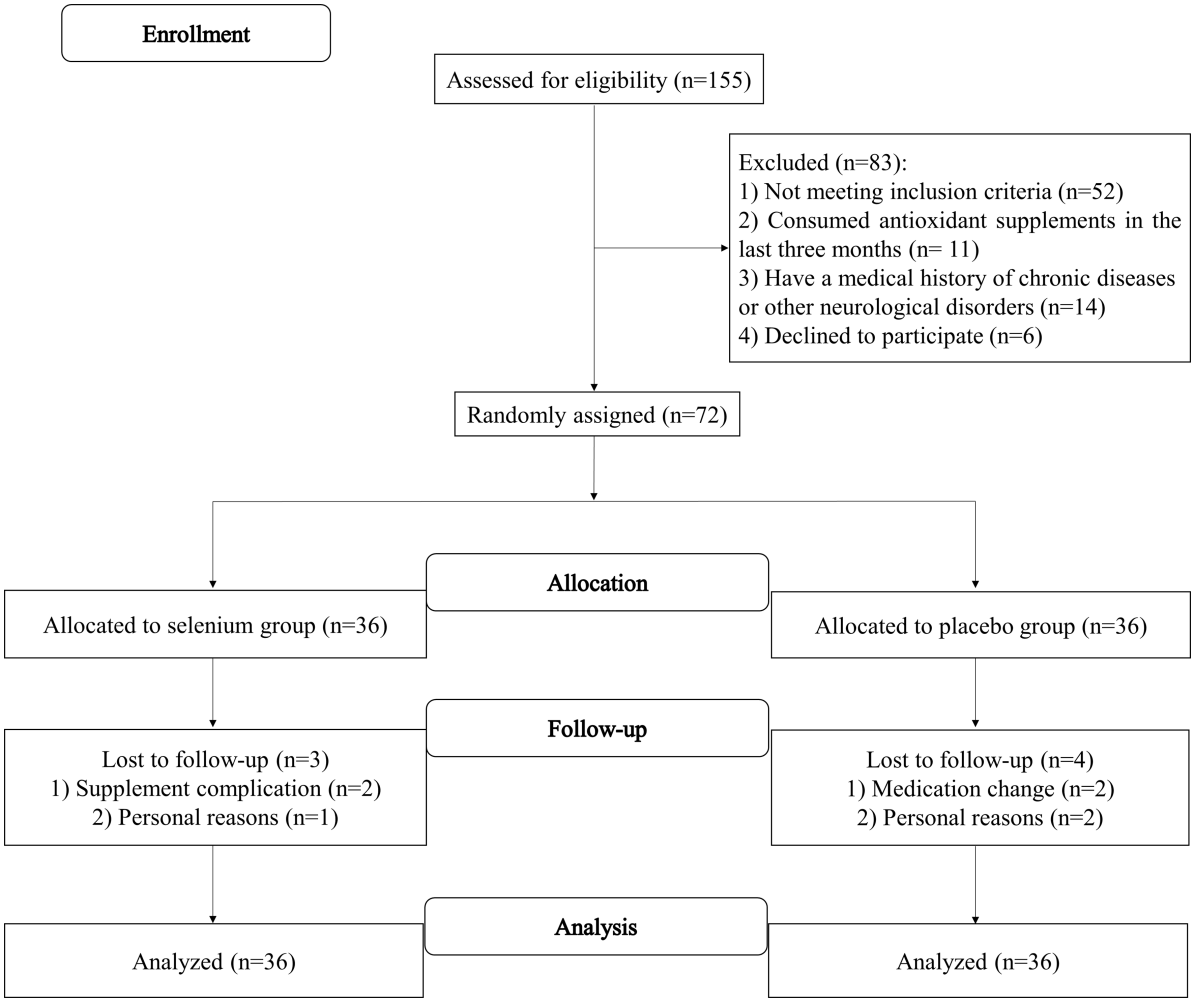
Flow diagram of the study recruitment.

The flow diagram of the recruitment process is shown in Figure 1. A total of 155 patients with migraine were initially screened according to the eligibility criteria. A total of 72 patients met the inclusion criteria and were randomly assigned to the selenium (*n* = 36) or placebo (*n* = 36) groups. From 72 subjects, three in the selenium group and four in the placebo group were excluded due to supplement complications (*n* = 2), medication change (*n* = 2), and personal reasons (*n* = 3). Finally, 33 patients in the selenium group and 32 patients in the placebo group had completed the trial. However, by using the ITT approach, measurements from all patients were included in the final analysis. The compliance rate of participants in the present study was 98%, which seems to be high.

### Descriptive data

The baseline characteristics of the study population in the selenium and placebo groups are provided in Table 1. Patients in the selenium group had significantly higher intakes of SNRIs than the placebo group. Moreover, individuals in the selenium group had significantly higher headache frequency and TOS. However, the total MSQ and EF scores in the selenium group were significantly lower than the controls (all *p*-values of <0.05). No other significant differences were observed between the two groups. Dietary intakes of the participants, throughout the trial, in the selenium and placebo groups are presented in Table 2. Participants in the selenium group had a higher intake of vitamin C compared to the placebo group (*p* = 0.02). There were no other between-group differences.

**Table 1:** Baseline characteristics of the study population in the selenium and placebo groups.

Variables			Selenium group (*n* = 36)^£^	Placebo group (*n* = 36)	*p*-value^*^
Demographic variables					
	Age (y)		40.83 ± 11.33	40.97 ± 11.27	0.96
	Female [number (%)]		29 (80.6)	29 (80.6)	0.99
	Married [number (%)]		29 (80.6)	30 (83.3)	0.76
	Diploma and higher [number (%)]		19 (52.8)	14 (39.0)	0.19
	Low socioeconomic status [number (%)]		10 (27.8)	18 (50.0)	0.13
	Weight (kg)		71.22 ± 10.97	68.39 ± 12.48	0.31
	BMI (kg/m^2^)		25.98 ± 3.38	25.59 ± 4.50	0.68
	WC (cm)		91.56 ± 9.65	89.53 ± 10.71	0.40
	Physical activity (METs/h/day)		2012.83 ± 306.59	1937.84 ± 349.51	0.34
	SBP (mmHg)		118.14 ± 16.62	116.92 ± 17.02	0.76
	DBP (mmHg)		82.61 ± 11.21	77.53 ± 11.73	0.06
	Migraine in first-degree relative [number (%)]		20 (55.6)	16 (44.4)	0.35
	Migraine with aura [number (%)]		29 (80.6)	27 (75.0)	0.57
	Chronic migraine [number (%)]		18 (50.0)	12 (33.3)	0.15
Drugs [number (%)]					
	Beta-blockers		32 (88.9)	30 (83.3)	0.50
	TCAs		23 (63.9)	29 (80.6)	0.11
	SNRIs		31 (86.1)	24 (67.0)	0.02
	Anticonvulsant		33 (91.7)	33 (91.7)	0.99
	Benzodiazepine		33 (91.7)	34 (94.4)	0.64
	Proton-pump inhibitor		34 (94.4)	35 (99.0)	0.15
Supplements [number (%)]					
	Magnesium		28 (77.8)	30 (83.3)	0.55
	Riboflavin		30 (83.3)	33 (91.7)	0.29
	Vitamin D		23 (63.9)	23 (63.9)	0.99
Outcomes					
	Frequency (day/mo)		15.92 ± 11.11	10.78 ± 8.32	0.03
	Duration (hour)		29.44 ± 22.54	22.81 ± 21.50	0.21
	Severity (VAS score)		8.00 ± 1.93	7.19 ± 2.51	0.13
	Total MSQ		50.11 ± 17.10	58.81 ± 14.99	0.03
		RR	24.22 ± 8.61	28.08 ± 7.42	0.07
		RP	15.00 ± 5.51	17.64 ± 5.55	0.07
		EF	10.89 ± 4.08	13.08 ± 3.55	0.02
	HIT-6		61.58 ± 8.05	59.64 ± 8.12	0.31
	Depression		13.89 ± 10.13	12.08 ± 12.90	0.51
	Anxiety		11.39 ± 7.68	9.06 ± 8.29	0.22
	Stress		19.11 ± 9.53	16.11 ± 10.43	0.21
	NO (nmol/ml)		11.15 ± 3.08	10.28 ± 3.11	0.24
	TAC (nmol of trolox equivalent/ml)		38.62 ± 13.22	39.27 ± 9.30	0.81
	TOS (nmol of H_2_O_2_ equivalent/ml)		21.87 ± 6.59	15.51 ± 5.00	<0.001
	MDA (nmol/ml)		21.73 ± 2.23	20.89 ± 1.66	0.08

Data are presented as mean ± SD or number (%).*Obtained from the chi-square analysis for categorical variables and the independent t-test for continuous variables.^£^Received daily tablet containing 200 μg of selenomethionine per day.BMI, body mass index; WC, waist circumference; SBP, systolic blood pressure; DBP, diastolic blood pressure; TCA, tricyclic antidepressants; SNRI, serotonin and norepinephrine reuptake inhibitors; VAS, visual analog scale; MSQ, migraine-specific quality of life; RR, role restrictive; RP, role preventive; EF, emotional functioning; HIT-6, headache Impact Test-6; NO, nitric oxide; TAC, total antioxidant capacity; TOS, total oxidant status; MDA, malondialdehyde; nmol, nanomoles; ml, milliliter; y, year; kg, kilogram; cm, centimeter; mmHg, millimeter of mercury.

**Table 2:** Dietary intakes of participants in the selenium and placebo groups.

Variables	Selenium group (*n* = 36)^£^	Placebo group (*n* = 36)	*p*-value^*^
Energy (Kcal/day)	1616.57 ± 554.13	1409.77 ± 363.22	0.07
Protein (g/day)	62.12 ± 22.14	56.81 ± 16.34	0.25
Fat (g/day)	46.77 ± 16.36	40.66 ± 17.70	0.13
Carbohydrate (g/day)	244.85 ± 99.54	209.87 ± 62.11	0.08
Selenium (mg/day)	0.01 ± 0.01	0.01 ± 0.01	0.34
Vitamin C (mg/day)	102.83 ± 48.80	76.53 ± 46.71	0.02
Vitamin E (mg/day)	4.46 ± 5.07	4.23 ± 4.64	0.84
Magnesium (mg/day)	160.27 ± 50.22	143.34 ± 49.60	0.16
Zinc (mg/day)	6.18 ± 2.26	5.60 ± 2.22	0.28
Iron (mg/day)	13.94 ± 7.96	11.51 ± 4.46	0.10
Copper (mg/day)	0.94 ± 0.43	0.79 ± 0.29	0.09
Folate (μg/day)	184.85 ± 85.90	157.88 ± 70.69	0.15
Riboflavin (mg/day)	0.83 ± 0.25	0.76 ± 0.25	0.25
Fiber (mg/day)	15.93 ± 5.66	13.66 ± 5.48	0.09

Data are presented as mean ± SD.*Obtained from the independent t-test.^£^Received daily tablet containing 200 μg of selenomethionine per day.Kcal, kilocalories; g, gram; mg, milligram; μg, microgram.

### Primary outcomes

#### Effect of selenium supplementation on oxidative stress

We found that selenium supplementation compared to placebo resulted in a significant reduction in serum TOS (−2.84 ± 10.23 vs. 0.31 ± 7.41, mean difference (MD) = −3.16 and 95% confidence interval (CI): −7.35, 1.04; *p* = 0.02) and NO levels (−1.68 ± 3.55 vs. 0.59 ± 3.11, MD = −2.27 and 95% CI: −3.84, −0.70; *p* = 0.01) and a significant increase in serum TAC (9.68 ± 15.07 vs. 0.04 ± 13.63, MD = 9.64 and 95% CI: 2.89, 16.40; *p* = 0.01). Moreover, selenium supplementation had a protective effect on MDA levels than placebo (0.34 ± 2.36 vs. 1.82 ± 3.85, MD = −1.48 and 95% CI: −2.98, 0.02; *p* = 0.04; Table 3). When the baseline values of outcome variables, energy and vitamin C intake, and SNRIs were adjusted in analyses, only the changes in TOS became non-significant (MD = 3.53 and 95% CI: −0.97, 8.03; *p* = 0.10; Table 4).

**Table 3:** The effect of selenium supplementation on clinical and physiological traits of migraine and oxidative stress biomarkers.

			Selenium group^§^ (*n* = 36)			Placebo group (*n* = 36)				
			Baseline	After	Mean change	Baseline	After	Mean change	Mean difference (95% CI)	*p*-value^£^
Clinical traits of migraine										
	Frequency (day/mo)		15.92 ± 11.11	6.11 ± 5.11	−9.80 ± 9.32	10.78 ± 8.32	8.31 ± 5.74	−2.47 ± 5.85	−7.33 (−10.99, −3.68)	<0.001
	Duration (hour)		29.44 ± 22.54	13.97 ± 15.97	−15.47 ± 24.27	22.81 ± 21.50	18.03 ± 15.28	−4.77 ± 24.68	−10.70 (−22.21, 0.81)	0.07
	Severity (VAS score)		8.00 ± 1.93	5.37 ± 1.97	−2.63 ± 2.42	7.19 ± 2.51	5.77 ± 2.30	−1.42 ± 2.55	−1.21 (−2.37, −0.04)	0.04
Quality of life										
	Total MSQ		50.11 ± 17.10	55.69 ± 15.39	5.58 ± 11.66	58.81 ± 14.99	59.43 ± 14.42	0.63 ± 12.61	4.95 (−0.76, 10.66)	0.09
		RR	24.22 ± 8.61	26.41 ± 9.30	2.19 ± 6.85	28.08 ± 7.42	26.83 ± 8.06	−1.25 ± 7.99	3.44 (−0.06, 6.94)	0.05
		RP	15.00 ± 5.51	17.86 ± 4.67	2.86 ± 5.38	17.64 ± 5.55	19.41 ± 5.08	1.77 ± 4.80	1.09 (−1.31, 3.48)	0.37
		EF	10.89 ± 4.08	11.42 ± 3.53	0.53 ± 2.36	13.08 ± 3.55	13.97 ± 3.48	0.88 ± 3.90	−0.36 (−1.87, 1.16)	0.64
	HIT-6		61.58 ± 8.05	52.26 ± 9.50	−9.33 ± 11.56	59.64 ± 8.12	57.67 ± 10.89	−1.97 ± 10.10	−7.35 (−12.66, −2.05)	0.01
Physiological traits										
	Depression		13.89 ± 10.13	12.23 ± 9.70	−1.66 ± 6.79	12.08 ± 12.90	10.87 ± 10.17	−1.22 ± 10.81	−0.44 (−4.69, 3.80)	0.52
	Anxiety		11.39 ± 7.68	10.34 ± 7.13	−1.05 ± 4.92	9.06 ± 8.29	9.27 ± 8.86	0.21 ± 5.63	−1.26 (−3.74, 1.23)	0.32
	Stress		19.11 ± 9.53	16.89 ± 7.89	−2.22 ± 6.70	16.11 ± 10.43	16.13 ± 9.49	0.02 ± 8.18	−2.24 (−5.77, 1.29)	0.21
Oxidative stress biomarkers										
	NO (nmol/ml)		11.15 ± 3.08	9.47 ± 2.71	−1.68 ± 3.55	10.28 ± 3.11	10.87 ± 2.44	0.59 ± 3.11	−2.27 (−3.84, −0.70)	0.01
	TAC (nmol of trolox equivalent/ml)		38.62 ± 13.22	48.29 ± 20.71	9.68 ± 15.07	39.27 ± 9.30	39.30 ± 10.07	0.04 ± 13.63	9.64 (2.89, 16.40)	0.01
	TOS (nmol of H_2_O_2_ equivalent/ml)		21.87 ± 6.59	19.02 ± 9.71	−2.84 ± 10.23	15.51 ± 4.50	15.82 ± 6.03	0.31 ± 7.41	−3.16 (−7.35, 1.04)	0.02
	MDA (nmol/ml)		21.73 ± 2.23	22.07 ± 2.19	0.34 ± 2.36	20.89 ± 1.66	22.71 ± 4.87	1.82 ± 3.85	−1.48 (−2.98, 0.02)	0.04

Values are expressed as mean ± SD or mean (95% CI).^£^Obtained from ANCOVA.^§^Received daily tablet containing 200 μg of selenomethionine per day.VAS, visual analog scale; HIT, headache-impact test; MSQ, migraine-specific quality of life; RR, role restrictive; RP, role preventive; EF, emotional functioning; NO, nitric oxide; TAC, total antioxidant capacity; TOS, total oxidative status; MDA, malondialdehyde; nmol, nanomol; ml, milliliter; mo, month; CI, confidence interval.

**Table 4:** Adjusted changes (95% CI) and differences (95% CI) of clinical and physiological traits of migraine and oxidative stress biomarkers.

			Selenium group^£^ (*n* = 36)	Placebo group (*n* = 36)	Mean difference (95% CI)	*p*-value^*^
Clinical traits of migraine						
	Frequency (day/mo)		−8.15 (−9.69, −6.61)	−4.12 (−5.66, −2.59)	−4.03 (−6.32, −1.73)	<0.001
	Duration (hour)		−14.99 (−23.58, −6.39)	−5.26 (−13.85, 3.34)	−9.73 (−22.36, 2.90)	0.13
	Severity (VAS score)		−2.89 (−3.73, −2.06)	−1.16 (−2.00, −0.32)	−1.73 (−2.96, −0.50)	0.01
Quality of life						
	Total MSQ		3.00 (−0.87, 6.84)	3.22 (−0.64, 7.07)	−0.22 (−6.09, 5.62)	0.94
		RR	2.05 (−0.57, 4.68)	−1.11 (−3.74, 1.51)	3.17 (−0.69, 7.02)	0.11
		RP	3.02 (1.27, 4.78)	1.61 (−0.15, 3.36)	1.42 (−1.16, 3.99)	0.28
		EF	0.45 (−0.66, 1.56)	0.96 (−0.15, 2.08)	−0.51 (−2.15, 1.12)	0.53
	HIT-6		−9.22 (−13.20, −5.23)	−2.08 (−6.06, 1.91)	−7.14 (−13.00, −1.28)	0.02
Physiological traits						
	Depression		−1.31 (−4.49, 1.87)	−1.56 (−4.74, 1.61)	0.25 (−4.42, 4.92)	0.53
	Anxiety		−0.65 (−2.49, 1.19)	−0.19 (−2.03, 1.65)	−0.46 (−3.16, 2.24)	0.74
	Stress		−2.22 (−4.88, 0.44)	−0.02 (−2.64, 2.69)	−2.24 (−6.16, 1.67)	0.26
Oxidative stress biomarkers						
	NO (nmol/ml)		−1.24 (−2.10, −0.38)	0.16 (−0.70, 1.02)	−1.40 (−2.67, −0.13)	0.03
	TAC (nmol of trolox equivalent/ml)		9.89 (4.91, 14.88)	−0.18 (−5.17, 4.81)	10.07 (2.74, 17.41)	0.01
	TOS (nmol of H_2_O_2_ equivalent /ml)		0.5 (−2.40, 3.40)	−3.03 (−5.92, −0.13)	3.53 (−0.97, 8.03)	0.10
	MDA (nmol/ml)		0.33 (−0.84, 1.48)	1.83 (0.68, 3.00)	−1.52 (−3.25, 0.22)	0.03

All values are presented as mean (95% CI) adjusted for energy intake, vitamin C, SNRIs, and baseline values (for outcomes with significant differences between the selenium and placebo groups at baseline).^£^Received daily tablet containing 200 μg of selenomethionine per day.*Obtained from ANCOVA.VAS, visual analog scale; HIT, headache-impact test; MSQ, migraine-specific quality of life; RR, role restrictive; RP, role preventive; EF, emotional functioning; NO, nitric oxide; TAC, total antioxidant capacity; TOS, total oxidative status; MDA, malondialdehyde; nmol, nanomol; ml, milliliter; mo, month; CI, confidence interval.

#### Effect of selenium supplementation on clinical traits of migraine

In comparison with placebo, selenium supplementation led to a lower severity (−2.63 ± 2.42 vs. −1.42 ± 2.55, MD = −1.21 and 95% CI: −2.37, −0.04; *p* = 0.04) and a lower frequency of migraine headaches (−9.80 ± 9.32 vs. −2.47 ± 5.85, MD = −7.33 and 95% CI: −10.99, −3.68; *p* < 0.001; Table 3). When the analyses were adjusted for baseline values of outcome variables, energy and vitamin C intake, and SNRIs, the severity (MD = −1.73 and 95% CI: −2.96, −0.50; *p* = 0.01) and frequency of headaches (MD = −4.03 and 95% CI: −6.32, −1.73; *p* < 0.001) between the selenium and placebo groups were also significantly different (Table 4).

### Secondary outcome

#### Effect of selenium supplementation on physiological traits and quality of life

Selenium supplementation had a significant beneficial effect on the quality of life (assessed by HIT-6) in comparison with the placebo group (−9.33 ± 11.56 vs. −1.97 ± 10.10, MD = −7.35 and 95% CI: −12.66, −2.05; *p* = 0.01). However, no significant effect was observed regarding psychological disorders (*p*-values>0.05; Table 3). The significant difference in HIT-6 scores between the selenium and placebo groups persisted after adjustment for baseline values of outcome variables, energy and vitamin C intake, and SNRIs (MD = −7.13 and 95% CI: −13.00, −1.26; *p* = 0.02; Table 4).

#### Effect of selenium supplementation on blood pressure and anthropometric parameters

No significant differences were observed regarding blood pressure and anthropometric indices between the selenium and placebo groups, either before or after adjustment for baseline values of variables, energy and vitamin C intake, and SNRIs (*p*-values>0.05; Tables 5, 6).

**Table 5:** The effect of selenium supplementation on blood pressure and anthropometric indices.

		Selenium group (*n* = 36)^§^			Placebo group (*n* = 36)				
		Baseline	After	Mean change	Baseline	After	Mean change	Mean difference (95% CI)	*p*-value^£^
Blood Pressure									
	SBP (mmHg)	118.14 ± 16.62	116.19 ± 16.51	−1.94 ± 9.30	116.92 ± 17.02	112.96 ± 13.18	−4.89 ± 16.33	2.95 (−3.63, 9.49)	0.39
	DBP (mmHg)	82.61 ± 11.21	81.82 ± 10.53	−1.33 ± 6.14	77.53 ± 11.73	76.25 ± 8.92	−3.07 ± 9.86	1.74 (−9.13, 3.91)	0.42
Anthropometric indices									
	BMI (kg/m^2^)	25.98 ± 3.37	26.14 ± 3.30	0.16 ± 0.60	25.59 ± 4.50	25.74 ± 4.25	0.15 ± 1.32	0.01 (−0.63, 0.91)	0.12
	WC (cm)	91.56 ± 9.65	91.83 ± 9.12	0.27 ± 2.64	89.53 ± 10.70	89.70 ± 10.73	−0.56 ± 1.69	0.83 (−0.60, 3.27)	0.05

Values are expressed as mean ± SD or mean (95% CI).^£^Obtained from ANCOVA.^§^Received daily tablet containing 200 μg of selenomethionine per day.SBP, systolic blood pressure; DBP, diastolic blood pressure; BMI, body mass index; WC, waist circumference; CI, confidence interval.

**Table 6:** Adjusted changes (95% CI) and differences (95% CI) of blood pressure and anthropometric indices.

		Selenium group (*n* = 36)^£^	Placebo group (*n* = 36)	Mean difference (95% CI)	*p*-value^*^
Blood pressure					
	SBP (mmHg)	−2.79 (−6.36, 0.78)	−5.62 (−9.19, −2.04)	2.83 (−2.42, 8.08)	0.29
	DBP (mmHg)	−6.39 (−9.37, −3.42)	−4.91 (−7.88, −1.93)	−1.49 (−5.88, 2.90)	0.50
Anthropometric indices					
	BMI (kg/m^2^)	0.26 (−0.26, 0.79)	0.18 (−0.34, 0.71)	0.08 (−0.69, 0.85)	0.84
	WC (cm)	0.36 (−0.99, 1.71)	−0.82 (−2.17, 0.53)	1.18 (−0.80, 3.16)	0.24

All values are presented as mean (95% CI) adjusted for energy intake, vitamin C, SNRIs, and baseline values (for outcomes with significant differences between the selenium and placebo groups at baseline).^£^Received daily tablet containing 200 μg of selenomethionine per day.*Obtained from ANCOVA.SBP, systolic blood pressure; DBP, diastolic blood pressure; BMI, body mass index; WC, waist circumference; CI, confidence interval.

### Side effects

During the study period, two patients reported nausea after taking selenium supplements; in these cases, intervention was immediately stopped. Other participants reported no serious side effects related to selenium supplementation.

## Discussion

In the present study, supplementation with 200 μg/day selenium for 12 weeks resulted in favorable changes in oxidative stress (TAC, NO, and MDA levels), frequency and severity of migraine headaches, and quality of life in patients with migraine compared to placebo. However, selenium had no significant effect on headache duration, psychological disorders, TOS levels, blood pressure, and anthropometric parameters. To the best of our knowledge, this was the first randomized controlled trial (RCT) to examine the effect of selenium supplementation on clinical and physiological outcomes of patients with migraine.

Despite the existence of many pharmacotherapies for the management of migraine, most of them are known to be ineffective and have potential side effects. For this reason, it is critical to look for safe and non-pharmacological approaches for the management of migraine headaches and improving patients’ quality of life. In the current RCT, selenium supplementation improved markers of oxidative stress including NO, MDA, and TAC levels in patients with migraine. However, no favorable effect was observed on TOS levels. Despite the lack of any investigation examining the effect of selenium on oxidative stress in migraineurs, this supplement has also been found to be helpful in other populations, such as gestational diabetes (31), polycystic ovary syndrome (22), and healthy individuals (22). In addition, our findings are in line with the study of Talaie et al., in which lower selenium levels in migraineurs were positively associated with MDA levels and the incidence of migraine attacks (6).

A growing body of evidence suggests that oxidative stress is involved in the pathogenesis of migraine (32, 33). NO, an oxidant vasodilator, is increased in the platelets, urine, and plasma of patients with migraine, which participates in trigeminovascular inflammation and also accelerates pain in the central nervous system (32). Moreover, elevated levels of lipid peroxidation metabolites such as MDA have been observed in migraineurs during headache attacks (12). For this reason, improvements observed regarding the frequency and severity of headaches can be explained by alleviating neurogenic inflammation and oxidative stress. Moreover, the current study indicated that selenium supplementation has a favorable effect on patients’ quality of life. A number of previous studies have investigated the effect of various antioxidants on migraine symptoms, of which, the results of a study by Kelishadi et al. showed that alpha-lipoic acid supplementation for 12 weeks had a reducing effect on the frequency and severity of headaches in women with episodic migraine (34). Another RCT revealed that curcumin and Q10 co-supplementation for 8 weeks improved the frequency, duration, and severity of headaches as well as quality of life in patients with episodic migraine (35). Such a beneficial effect was also reported for zinc, vitamin C, E, and N-acetylcysteine (36, 37). The conflicting results may be partially explained by the administration of different antioxidants and covariates considered in the analyses. The beneficial effect of selenium on migraine symptoms may be justified by some potential mechanisms. Low activity of glutathione peroxidase (a selenium-dependent enzyme) is documented in patients with migraine, and its reduction attenuates antioxidant defenses and causes neuroinflammation (38, 39). Additionally, recent reports suggest that selenium could manage the pain in the central nervous system by inhibiting apoptosis and mitochondrial oxidative stress and modulating Ca^2+^ influx through cation channels such as transient receptor potential (TRP) melastatin 2 (TRPM2) (40, 41).

In the current study, unlike the frequency and severity of headaches, selenium had no effect on headache duration. Previous studies have shown that headache duration has a late response to interventions designed for patients with migraine (16, 34). Therefore, a longer duration of selenium treatment and intervention with higher doses may affect the duration of headaches in migraineurs. In addition, it is possible that reporting bias is higher in headache duration than headache frequency.

In this survey, selenium supplementation had no significant effect on physiological disorders including depression, anxiety, and stress. In contrast to our findings, a recent clinical trial reported that supplementation with alpha-lipoic acid, as an antioxidant, led to a significant reduction in depression, anxiety, and stress scores in patients with migraine (42). The observed disparity between our findings and the previous study might be due to the differences in the studied population and the administration of different antioxidants. Moreover, in the current RCT, selenium supplementation had no effect on blood pressure or anthropometric indices, which was in line with prior investigations on other populations (31, 43, 44).

### Strengths and limitations

The present study was the first RCT evaluating the effect of selenium supplementation on migraine symptoms, mental disorders, and biomarkers of oxidative stress among patients with migraine. Patients and investigators in the current study were blinded to the prescribed interventions. Dietary intakes throughout the trial were monitored, and the differences were adjusted. However, there are some limitations that should be taken into account. Despite using validated questionnaires for the assessment of outcome variables, the misclassification bias cannot be completely ruled out. In addition, due to limited financial resources, we were not able to measure serum levels of selenium, inflammatory biomarkers, and more oxidative stress indicators. As the assessment of selenium levels is critical to determine the extent of selenium deficiency in patients and ensure accurate compliance rates, future RCTs should consider this issue. Although the serum level of oxidative stress biomarkers was alleviated in this study, it should be noted that these values do not reflect the cerebrospinal fluid changes in patients. Finally, due to the study’s small sample size, we were unable to conduct subgroup analyses based on sex, types of migraine, etc., and also unable to adjust more confounders (e.g., medications, supplements, and physical activity) in the analyses. Therefore, there is a need for further large-scale studies to replicate these findings.

## Conclusion

We found that 12-week selenium supplementation had beneficial effects on oxidative stress biomarkers of NO, MDA, and TAC in patients with migraine. Moreover, improvements were observed regarding the frequency and severity of migraine headaches as well as the HIT-6 score. However, we found no significant effect on psychological disorders or the duration of migraine headaches. Further clinical trials should be designed to replicate our findings and discover an appropriate dosage.

## Data availability statement

The datasets presented in this article are not readily available because Data described in the manuscript will be made available upon reasonable request by the corresponding author. Requests to access the datasets should be directed to askari@mui.ac.ir.

## Ethics statement

The studies involving humans were approved by Ethic committee of Isfahan University of Medical Sciences. The studies were conducted in accordance with the local legislation and institutional requirements. The participants provided their written informed consent to participate in this study.

## Author contributions

AB: Investigation, Writing – original draft. OS: Conceptualization, Writing – original draft. FK: Conceptualization, Writing – original draft. MR: Conceptualization, Writing – original draft. GA: Conceptualization, Methodology, Writing – original draft.

## Footnotes

^1^ https://www.irct.ir

^2^ https://www.sealedenvelope.com/simple
